# Potential Contribution of Age-Related Soft-Tissue Changes to Delayed Contour Visibility After Autologous Rhinoplasty: A Narrative Review

**DOI:** 10.3390/medicina62091660

**Published:** 2026-08-29

**Authors:** Eojina Lee, Soyoung Ko, Chul Chang

**Affiliations:** Department of Otolaryngology-Head & Neck Surgery, Veterans Health Service Medical Center, 53, Jinhwangdo-ro 61-gil, Gangdong-gu, Seoul 05368, Republic of Korea; jina1107714@naver.com (E.L.); ko.soyoung312@gmail.com (S.K.)

**Keywords:** rhinoplasty, aging, thin nasal skin, autologous cartilage graft, contour irregularity, soft-tissue aging

## Abstract

*Background and Objectives*: Delayed contour irregularities may become apparent years after otherwise successful autologous rhinoplasty, particularly in patients with thin nasal skin. Thin-skinned rhinoplasty, autologous graft behavior, and nasal aging have been studied individually, but their potential interaction remains unclear. This review examines current evidence on age-related changes in the nasal soft-tissue envelope, thin nasal skin, autologous graft behavior, and their potential effects on long-term rhinoplasty outcomes. *Materials and Methods*: A structured narrative review was conducted using PubMed as the primary database, with supplementary searches of Google Scholar and reference lists. Four predefined search domains addressed thin nasal skin, nasal aging, autologous graft behavior, and preservation and camouflage techniques. The PubMed searches identified 463 records. After duplicate removal and title and abstract screening, 205 publications were retained for further assessment. Additional relevant publications were identified through supplementary searches. Evidence was synthesized qualitatively because of differences in study design, surgical technique, follow-up duration, and outcome assessment. *Results*: Thin nasal skin is associated with increased visibility of underlying structural features after rhinoplasty. Aging is associated with changes in skin thickness and elasticity, soft-tissue volume, and the underlying nasal framework. These findings provide a possible basis for changes in soft-tissue camouflage over time, but direct longitudinal evidence linking aging to delayed graft visibility is lacking. Late contour changes may also result from graft warping or resorption, scar remodeling, or changes in the native nasal framework. The ability of preservation and camouflage techniques to prevent aging-related delayed contour visibility has not been established. *Conclusions*: Age-related changes in the nasal soft-tissue envelope may contribute to delayed contour visibility after rhinoplasty. However, direct longitudinal evidence showing that these changes increase the visibility of an otherwise stable autologous graft is lacking. This relationship should therefore be considered a hypothesis rather than an established causal mechanism. Prospective longitudinal studies using objective measurements of soft-tissue thickness, graft morphology, and external nasal contour are needed to test this hypothesis.

## 1. Introduction

Autologous cartilage is widely used in structural rhinoplasty because of its biocompatibility and favorable long-term outcomes [1,2]. Septal, auricular, and costal cartilage grafts are commonly used to restore nasal contour and provide structural support [1,3,4]. Delayed contour irregularities, graft visibility, and subtle asymmetries may occur, particularly in patients with thin nasal skin [5,6,7].

Thin nasal skin provides limited soft-tissue coverage over the underlying framework [5,6,7,8]. Minor graft edges, contour irregularities, or surface asymmetries may therefore be more visible in patients with thin skin. Nasal soft-tissue thickness also varies according to anatomical site [9,10,11]. Graft refinement, soft-tissue preservation, and camouflage techniques are important considerations in thin-skinned patients [12,13].

The nasal soft-tissue envelope and supporting structures change with aging [14,15,16,17,18,19,20,21,22,23]. Age-related changes involve skin thickness and elasticity, soft-tissue volume, and the underlying nasal framework [16,18,19,20,21,22,23]. Nasal ligaments also contribute to structural support [24]. Such changes could affect the long-term appearance of reconstructed nasal structures. However, direct evidence showing that age-related soft-tissue changes increase the visibility of an otherwise stable autologous graft is lacking.

Thin nasal skin, autologous graft behavior, and nasal aging have been studied separately. Their interaction over time is less well defined. Late contour changes may result from graft-related factors, changes in the native nasal framework, postoperative tissue remodeling, or changes in the overlying soft-tissue envelope. The relative contribution of these factors remains unclear.

**Figure 1:**
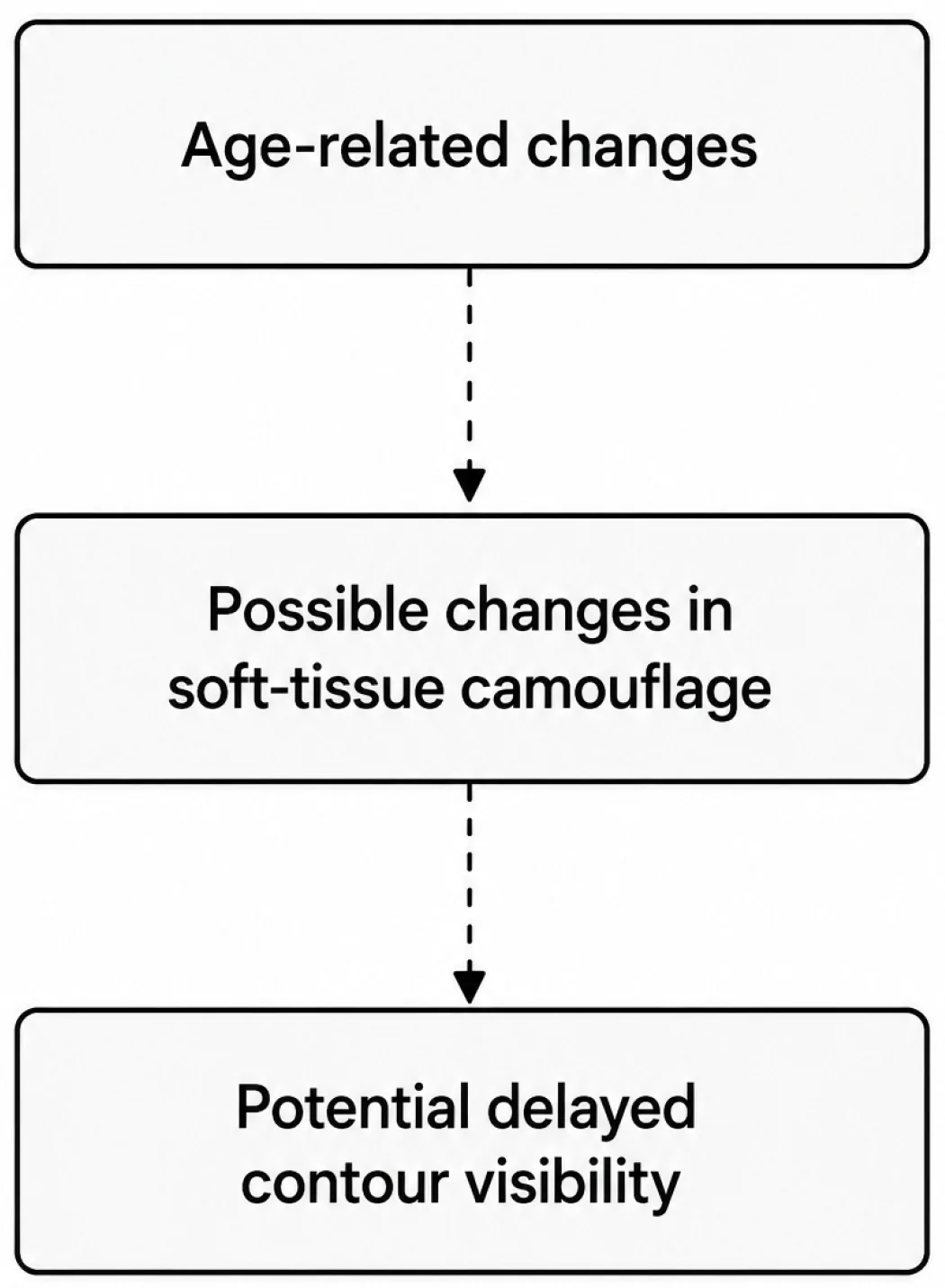
Hypothesized framework for delayed contour visibility after autologous rhinoplasty. Dashed arrows indicate proposed rather than established relationships. Age-related changes may alter soft-tissue camouflage and potentially contribute to delayed contour visibility.

This narrative review examines the available evidence on thin nasal skin, age-related changes in the nasal soft-tissue envelope, autologous graft behavior, and long-term contour outcomes after rhinoplasty. It also considers the possible contribution of soft-tissue aging to delayed contour visibility and identifies areas that require further study (Figure 1).

### 1.1. Literature Search

This study was conducted as a structured narrative review. PubMed was used as the primary database and was searched from database inception through 30 June 2026. Four search domains were used: (1) thin nasal skin and contour visibility, (2) nasal aging and rhinoplasty, (3) autologous grafts and long-term contour outcomes, and (4) preservation and camouflage techniques. The search terms included “rhinoplasty”, “thin nasal skin”, “nasal skin thickness”, “soft-tissue envelope”, “nasal aging”, “aging nose”, “autologous cartilage”, “cartilage graft”, “contour irregularity”, “graft visibility”, “warping”, “resorption”, “preservation rhinoplasty”, “fascia”, “diced cartilage”, and “camouflage”. The PubMed search domains, search terms, and numbers of records retrieved are provided in Supplementary Table S1.

The four PubMed searches identified 21, 133, 105, and 204 records, respectively, for a total of 463 records. After removal of 47 duplicate records, 416 unique records remained. Google Scholar and the reference lists of relevant articles were used as supplementary sources to identify additional relevant or landmark publications. These supplementary sources were not included in the PubMed record count.

### 1.2. Eligibility and Study Selection

Studies were considered eligible if they addressed at least one of the following topics: age-related changes in nasal anatomy or soft tissues; thin nasal skin or nasal soft-tissue thickness; long-term behavior of autologous grafts, including resorption, warping, displacement, or contour visibility; delayed contour changes after rhinoplasty; or camouflage and preservation techniques relevant to contour visibility. Clinical studies, anatomical studies, systematic reviews, narrative reviews, and relevant technical articles were considered.

Studies were excluded if they were unrelated to rhinoplasty, nasal aging, graft behavior, or nasal soft tissues. Studies limited to cleft, traumatic, oncologic, or non-nasal reconstruction without findings relevant to the review question were also excluded. Duplicate publications and technical reports without relevant outcome or anatomical information were excluded.

Of the 416 unique records, 123 were excluded after title screening. The remaining 293 records underwent title and abstract screening, and 205 were retained for further assessment. Screening was performed by one reviewer using the predefined criteria. Because this was a narrative review, no formal systematic full-text eligibility stage was performed after this screening step. Publications were subsequently selected from this pool according to their relevance to the predefined review domains, with additional relevant or landmark publications identified through Google Scholar and reference-list screening. The final narrative synthesis cited 38 publications.

### 1.3. Definitions and Evidence Synthesis

Delayed contour irregularities were defined as contour changes that developed or became more apparent after an initially satisfactory postoperative result. Because no widely accepted time threshold was identified, a fixed cutoff for “delayed” or “long-term” was not used.

Thin nasal skin was interpreted according to the definition or assessment method used in each study. Studies based on clinical assessment and those using objective measurements of nasal soft-tissue thickness were both considered. Differences in thickness among nasal anatomical sites were considered when interpreting the findings.

The evidence was synthesized qualitatively because the included publications differed in study design, patient population, surgical technique, graft material, follow-up duration, and outcome assessment. No meta-analysis was performed.

Evidence in Table 1, Table 2 and Table 3 was classified as consistent, moderately consistent, or limited. Consistent evidence indicated similar findings across multiple relevant studies. Moderately consistent evidence indicated generally similar findings with limitations in study number, design, or follow-up. Limited evidence indicated mainly indirect evidence, small or retrospective studies, technical reports, or a lack of direct long-term clinical data. These categories were used for narrative interpretation and do not represent a formal grading system. These categories describe only the direction and apparent agreement of findings across the available literature and do not indicate methodological quality, certainty of evidence, or risk of bias.

## 2. Long-Term Behavior of Autologous Grafts

Autologous cartilage is widely used in rhinoplasty because of its biocompatibility and low risk of extrusion [1]. Septal, auricular, and costal cartilage are commonly used according to the amount and structural support required [1,3,4]. Costal cartilage provides abundant material and strong support but may be associated with warping and resorption [3].

Autologous cartilage generally has favorable long-term outcomes, but complications can occur. Reported complications include warping, resorption, infection, and contour irregularity [2,3]. Complication rates vary among studies because of differences in graft material, surgical technique, follow-up duration, and outcome definitions [3]. Long-term data extending over many years remain limited.

Late contour changes may result from the graft itself or from other postoperative changes. Graft warping and resorption may alter nasal contour [2,3]. Scar remodeling and changes in the surrounding soft tissues may also affect the external contour. These mechanisms may occur separately or together.

Direct evidence linking age-related changes in the nasal soft-tissue envelope to increased visibility of an otherwise stable graft is limited. Late graft visibility should therefore be evaluated together with structural graft changes and other possible causes. This distinction is important when evaluating delayed contour irregularities (Table 1).

## 3. Thin Nasal Skin and Contour Visibility

Thin nasal skin provides less coverage of the underlying nasal framework and may increase the visibility of graft edges, contour irregularities, and surface asymmetries [5,6,7,12]. Careful graft shaping and soft-tissue management are important in patients with thin skin [12,13].

Thin nasal skin is not defined uniformly in the literature. Some studies use clinical examination or surgeon assessment, whereas others use objective measurements of the nasal soft-tissue envelope [9,10,11]. Soft-tissue thickness also varies among nasal regions [9,10,11]. These differences limit the use of a single definition of thin nasal skin.

Camouflage techniques used in thin-skinned patients include fascia and diced cartilage-based methods [12,13,27,28,29]. These techniques may reduce the visibility of underlying structural irregularities. However, the studies differ in technique, follow-up duration, and outcome assessment, and direct comparative evidence is limited [12,13,27,28,29,30].

Thin skin alone does not explain all late contour irregularities. Graft material and position, scar remodeling, surgical technique, changes in the native framework, and age-related changes in the overlying soft tissues may also contribute. Direct longitudinal studies examining the interaction between thin skin, aging, and delayed graft visibility are lacking.

## 4. Age-Related Changes in the Nasal Soft-Tissue Envelope

Aging affects the skin, soft tissues, and supporting structures of the nose [14,15,16,17,18,19,20,21,22,23]. Age-related skin changes include alterations in dermal thickness, collagen and elastin characteristics, and skin elasticity [18,19,23]. Changes in facial soft-tissue volume also occur with aging [20]. These processes are distinct and should not be considered simply as skin thinning.

Nasal ligaments contribute to tip support and stability [24]. Age-related skeletal remodeling of the nasal and perinasal region has also been described [16,21]. Clinical studies of the aging nose have reported changes in nasal length, tip position, and the nasolabial angle [17,22].

These findings show that nasal anatomy changes with age. However, most studies do not examine patients longitudinally after rhinoplasty. Direct measurements of postoperative changes in nasal skin thickness, soft-tissue volume, and graft visibility over many years are limited.

Age-related skin changes vary among individuals. Intrinsic aging and external factors, including sun exposure, can affect skin characteristics [18,19]. Age alone may therefore not predict changes in the nasal soft-tissue envelope or long-term contour appearance.

**Figure 2:**
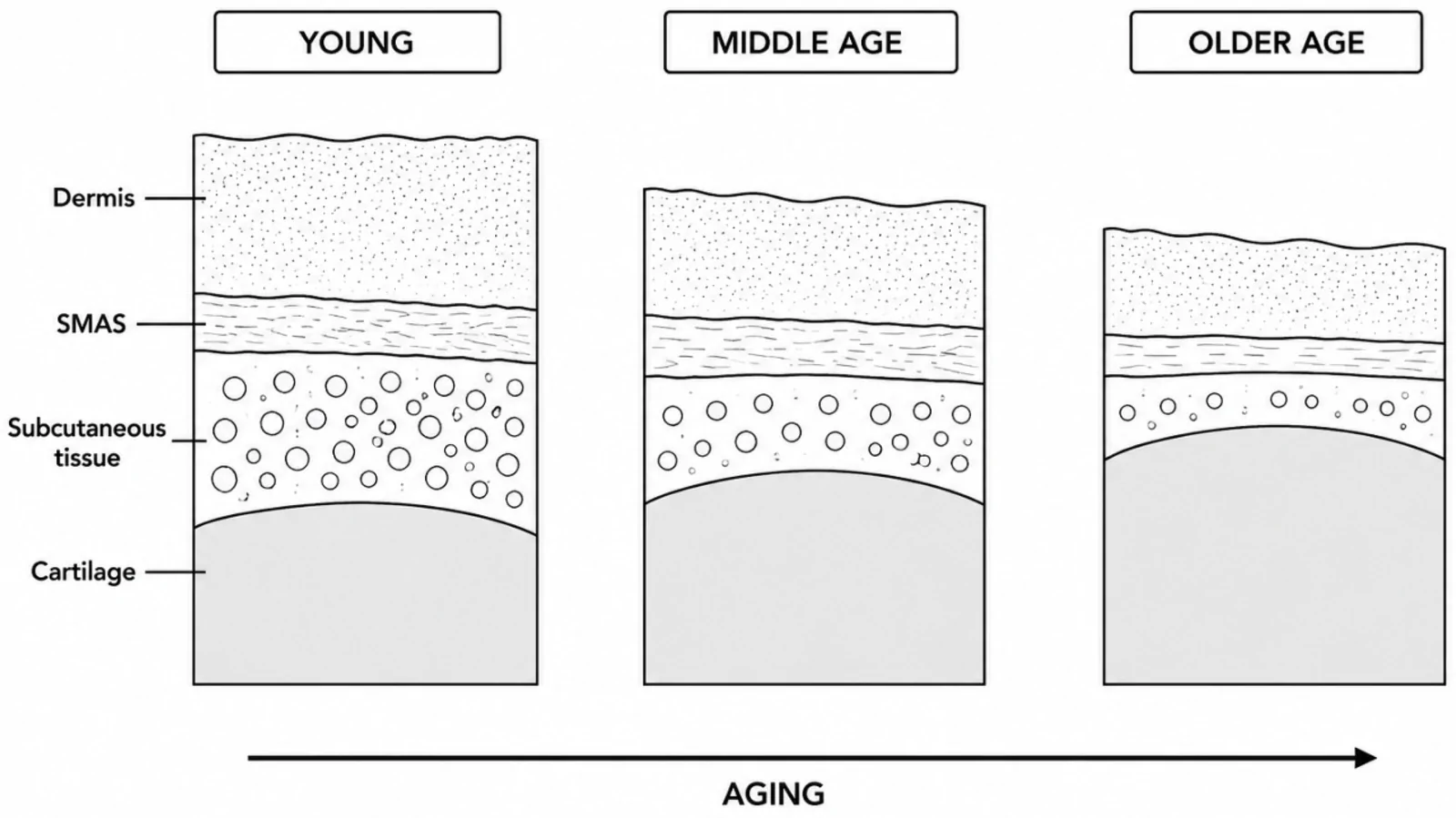
Age-related changes in the nasal soft-tissue envelope. Changes may involve the skin, subcutaneous tissues, supporting soft-tissue layers, and the underlying nasal framework. The pattern and extent of these changes may vary among individuals. The arrow indicates the direction of increasing age.

Available evidence shows that the nasal skin, soft tissues, and supporting framework change with aging. Whether these changes affect long-term graft visibility remains uncertain (Figure 2).

## 5. Potential Interaction Between Soft-Tissue Aging and Graft Visibility

Long-term contour after rhinoplasty is influenced by both the underlying framework and the overlying soft-tissue envelope. Autologous grafts may remain stable, but structural changes such as resorption and warping can occur over time [2,3]. These changes should be considered when evaluating late contour irregularities.

Aging is associated with changes in skin thickness and elasticity, soft-tissue volume, and the underlying nasal framework [16,18,19,20,21,22,23]. Nasal ligaments also contribute to structural support [24]. Thin soft-tissue coverage is associated with greater visibility of underlying structural features [5,6,7,9,10,11,12]. Based on these separate observations, it is possible that age-related changes in the soft-tissue envelope could make a previously subtle graft contour more visible over time.

This proposed mechanism should be distinguished from structural change in the graft itself. Late contour irregularities may result from graft warping or resorption, scar remodeling, changes in the native nasal framework, or changes in the overlying soft tissues [2,3,16,21]. More than one mechanism may be present in the same patient. Broader rhinoplasty practice data also show that secondary procedures remain a relevant part of clinical practice [38].

**Figure 3:**
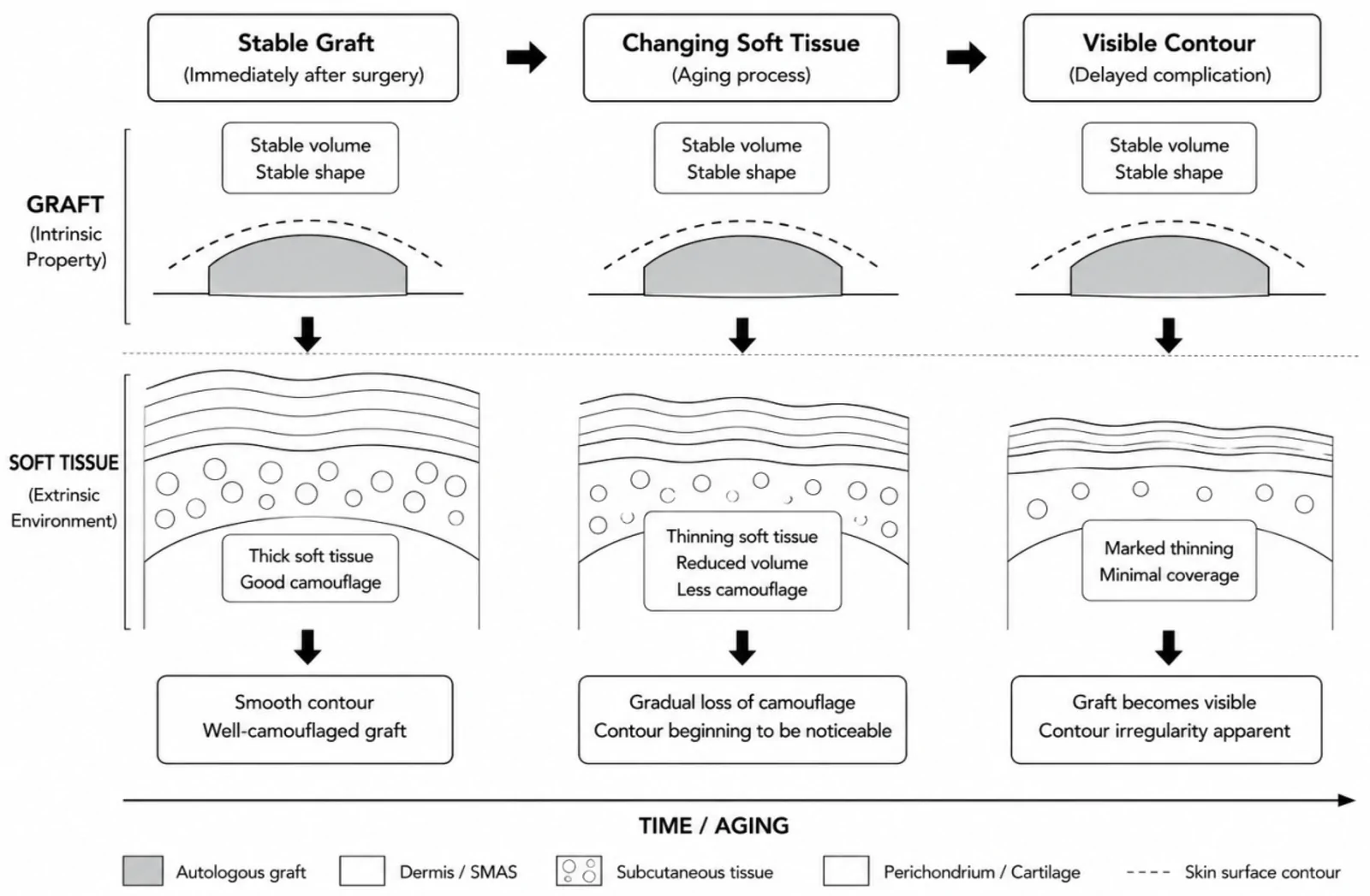
Proposed interaction between age-related soft-tissue changes and graft visibility. Changes in the nasal soft-tissue envelope may alter the visibility of an otherwise stable graft over time. This is a conceptual model; a causal relationship has not been established by longitudinal clinical studies.

Current studies do not establish that age-related soft-tissue changes cause increased visibility of an otherwise stable graft. Direct longitudinal studies measuring both graft structure and the overlying soft-tissue envelope are lacking. The proposed interaction between soft-tissue aging and graft visibility should therefore be considered a hypothesis rather than an established causal relationship (Figure 3).

Longitudinal studies using objective measurements of nasal soft-tissue thickness, graft morphology, and external contour are needed to test this hypothesis. Serial photography and three-dimensional imaging may also be useful for evaluating changes over time [37].

## 6. Preservation and Camouflage Strategies

Preservation rhinoplasty includes techniques that aim to maintain native nasal structures while limiting disruption of the dorsal framework and surrounding soft tissues [32,33]. A systematic review and meta-analysis found fewer dorsal irregularities with dorsal preservation than with dorsal reduction, although residual or recurrent hump remained a potential complication [34]. Long-term outcomes after dorsal preservation have also been reported [35].

Camouflage techniques may be useful in patients with thin soft-tissue coverage. Reported techniques include fascia lata, temporalis fascia, deep temporal fascia, and diced cartilage-based methods [13,27,28,29]. Other approaches to dorsal augmentation have also been described [30]. Clinical studies have reported favorable contour outcomes with these techniques in selected patients, although direct comparisons are limited [12,13,27,28,29].

Careful graft preparation and positioning are important in patients with thin skin [1,12]. Potential limitations vary according to the material and technique. Diced cartilage has been associated with complications such as visible irregularity, absorption, and infection [25], while partial resorption has been reported withAlloDerm [31].

Current evidence does not show that preservation or camouflage techniques prevent aging-related delayed contour visibility. These techniques may be considered when clinically and anatomically appropriate, but their role in preventing late changes related to aging has not been established (Table 2).

Longer follow-up with standardized photography and objective soft-tissue measurements may help define the long-term effects of these techniques. Three-dimensional imaging may provide an additional method for objective contour assessment [37].

## 7. Current Evidence Gaps and Future Research

Few studies have examined the long-term interaction among nasal soft-tissue changes, graft behavior, and contour visibility after rhinoplasty.

Available studies vary in design and often have limited long-term follow-up. Study populations, graft materials, surgical techniques, follow-up periods, and outcome measures also vary. These differences limit comparison across studies. Objective measurements of postoperative soft-tissue thickness and graft morphology are also rarely reported.

Future studies should use prospective longitudinal designs. Patients could be grouped according to baseline soft-tissue thickness, age, graft material, graft location, and surgical technique. Comparison of patients with thinner and thicker soft-tissue envelopes may help determine whether baseline tissue thickness is associated with late contour visibility.

Serial assessment should include standardized photography and objective measurements of the nasal soft-tissue envelope. Three-dimensional surface imaging may be useful for measuring changes in external nasal contour over time [37]. When feasible, imaging methods that can assess both soft-tissue thickness and graft morphology could help distinguish changes in the graft from changes in the overlying tissues.

Follow-up over several years may be needed to evaluate delayed changes. Outcome assessment could include objective contour measurements, photographic assessment, patient-reported outcomes, and revision surgery. These studies may help determine whether aging-related soft-tissue changes independently contribute to delayed contour visibility after rhinoplasty (Table 3).

## 8. Conclusions

Thin nasal skin provides limited camouflage of underlying structural features, and the nasal soft-tissue envelope and supporting framework change with aging. These findings provide a basis for the hypothesis that age-related soft-tissue changes may contribute to delayed contour visibility after rhinoplasty.

The proposed relationship between soft-tissue aging and delayed graft visibility remains a hypothesis, and alternative mechanisms such as graft warping or resorption, scar remodeling, and changes in the native nasal framework should also be considered.

Prospective longitudinal studies using objective measurements of soft-tissue thickness, graft morphology, and external nasal contour are needed to test this hypothesis.

## Figures and Tables

**Table 1:** Summary of Evidence Relevant to the Proposed Relationship Between Aging and Delayed Contour Visibility After Rhinoplasty.

Evidence Domain	Main Finding	Evidence Type	Evidence Consistency	Key References
Thin nasal skin and contour visibility	Reduced soft-tissue coverage increases visibility of contour irregularities.	Direct clinical and anatomical evidence (A/B)	Consistent	[5,6,7,8,9,10,11,12]
Objective nasal soft-tissue thickness	Nasal soft-tissue thickness varies by anatomical subsite and patient characteristics.	Anatomical/imaging evidence (B)	Consistent	[9,10,11]
Aging-related skin changes	Aging changes skin thickness, elasticity and mechanical properties.	Indirect aging-related evidence (B)	Consistent	[18,19,23]
Aging-related nasal structural changes	Nasal soft tissues and supporting structures change with aging.	Anatomical/imaging and clinical evidence (A/B)	Consistent	[14,15,16,17,21,22,24]
Long-term autologous graft behavior	Autologous grafts may undergo long-term structural changes.	Direct clinical/technical evidence (A/C)	Moderately consistent	[2,3,4,25,26]
Camouflage techniques	Camouflage techniques may reduce visibility of contour irregularities.	Clinical/technical evidence (A/C)	Moderately consistent	[12,13,27,28,29,30,31]
Preservation rhinoplasty	Preservation techniques may maintain native structural relationships.	Clinical/technical evidence (A/C)	Moderately consistent	[32,33,34,35]
Aging-related loss of camouflage causing delayed visibility of a stable graft	Biologically plausible, but lacking direct longitudinal evidence.	Indirect evidence/proposed mechanism (B/D)	Limited	Indirectly supported by [9,10,11,12,14,15,16,17,18,19,20,21,22,23,24]

Note: Evidence domains were classified as follows: A, direct clinical evidence from rhinoplasty populations; B, indirect anatomical or aging-related evidence; C, technical evidence concerning graft behavior, camouflage, or preservation; and D, contextual evidence from reviews, expert literature, and methodological studies. Evidence consistency was categorized as consistent, moderately consistent, or limited according to the qualitative criteria described in Section 1.3. Evidence consistency refers to the apparent agreement of findings across the reviewed literature and should not be interpreted as a measure of evidence quality or certainty. These categories do not represent a formal evidence-grading system.

**Table 2:** Clinical Implications and Cautious Considerations for Long-Term Contour Management.

Clinical Consideration	Potential Rationale	Current Evidence	Suggested Interpretation
Preoperative assessment of the soft-tissue envelope	Reduced soft-tissue coverage may increase visibility of contour irregularities.	Consistent [5,6,7,8,9,10,11,12]	Consider soft tissue characteristics when planning structural grafting.
Careful graft contouring and positioning	Graft changes may contribute to late contour irregularities.	Moderately consistent [1,2,3,4,26]	Careful graft preparation and placement remain important.
Selective use of camouflage materials	Fascia, diced cartilage or other coverage techniques may improve contour smoothness.	Moderately consistent [12,13,27,28,29,30,31]	Consider camouflage in patients with limited soft-tissue coverage.
Consideration of preservation techniques	Preservation techniques may reduce disruption of native structural relationships.	Moderately consistent [32,33,34,35]	Consider preservation techniques when anatomically appropriate; benefit for delayed contour visibility is unproven.
Counseling regarding long-term contour change	Both graft- and patient-related factors may affect contour irregularities over time.	Moderately consistent [2,3,14,15,16,17,21,22,25,36]	Patients may be informed that late contour change is multifactorial.
Long-term clinical follow-up	Delayed changes may emerge over time.	Limited	Longer follow-up may be useful in selected high-risk patients.
Objective documentation	Serial assessment may help distinguish structural from soft-tissue changes.	Limited/emerging [9,10,11,37]	Use standardized photography and objective measurements for long-term assessment.

Note: These statements are intended as careful clinical considerations rather than evidence-based treatment guidelines. In particular, current evidence does not establish that camouflage or preservation techniques prevent aging-related delayed contour visibility.

**Table 3:** Differential Mechanisms of Late Contour Irregularities After Rhinoplasty.

Potential Mechanism	Primary Structure Affected	Possible Clinical Manifestation	Supporting Evidence/Context	Relationship to Aging Hypothesis
Graft warping/deformation	Autologous graft	Altered contour	Direct graft-related evidence [3,26]	Alternative mechanism
Graft resorption	Autologous graft	Altered contour	Direct clinical evidence [2,3]	Alternative mechanism
Graft displacement/migration	Graft–host interface	Altered contour	Clinical/technical evidence [3,4,26,36]	Alternative mechanism
Diced-cartilage–related change	Diced cartilage construct	Irregularity, displacement	Meta-analytic/clinical evidence [25]	Alternative mechanism
Scar contraction/remodeling	Soft tissue/scar	Retraction, Irregularity	Clinical/technical context [36]	May coexist with aging
Infection or chronic inflammation	Graft/surrounding tissues	Altered contour	Rhinoplasty complication evidence [3,25,36]	Alternative mechanism
Native framework remodeling	Bone/cartilage	Altered contour	Anatomical/aging evidence [14,15,16,17,21,22,24]	May be aging-related but distinct from soft-tissue camouflage
Soft-tissue volume change	Soft-tissue envelope	Altered soft-tissue coverage	Indirect aging evidence [18,19,20,21,23]	Biologically supports proposed mechanism
Age-related skin change	Skin/dermis	Altered camouflage	Indirect aging evidence [18,19,23]	Biologically supports proposed mechanism
Trauma, weight change, or subsequent procedures	Variable	Altered contour	Clinical contextual factors	Confounding/alternative mechanism
Stable graft with altered overlying camouflage	Soft-tissue envelope over stable graft	Increased graft visibility	No direct longitudinal validation	Proposed hypothesis

Note: The proposed mechanism of increased visibility of an otherwise stable graft secondary to altered soft-tissue camouflage is distinguished from structural graft changes and other alternative causes. The proposed aging-related mechanism remains hypothesis-generating because direct longitudinal clinical validation is lacking.

## Data Availability

No new data were created or analyzed in this study. Data sharing is not applicable to this article.

## Supplementary Materials

The following supporting information can be downloaded at: https://www.mdpi.com/article/10.3390/medicina62091660/s1, Table S1: Reconstructed PubMed Boolean Search Strategies.
