# Supplementary material for: Potential Contribution of Age-Related Soft-Tissue Changes to Delayed Contour Visibility After Autologous Rhinoplasty: A Narrative Review
Source: Medicina (Kaunas). 2026 Aug 29;62(9):1660. doi: 10.3390/medicina62091660 (PMC13609260; doi:10.3390/medicina62091660)
Supplement: Supplementary file 1 [file medicina-62-01660-s001.zip › medicina-4497711-supplementary.pdf]

Supplementary Table S1. Reconstructed PubMed Boolean Search Strategies

Search domain	Reconstructed PubMed Boolean strategy	Original records retrieved
1. Thin nasal skin and contour visibility	("rhinoplasty"[Title/Abstract] OR "nasal surgery"[Title/Abstract]) AND ("thin skin"[Title/Abstract] OR "thin nasal skin"[Title/Abstract] OR "nasal skin thickness"[Title/Abstract] OR "soft tissue envelope"[Title/Abstract] OR "soft-tissue envelope"[Title/Abstract]) AND ("contour irregularity"[Title/Abstract] OR "contour irregularities"[Title/Abstract] OR "graft visibility"[Title/Abstract] OR "contour visibility"[Title/Abstract] OR camouflage[Title/Abstract])	21
2. Nasal aging and rhinoplasty	("rhinoplasty"[Title/Abstract] OR "nasal surgery"[Title/Abstract] OR nose[Title/Abstract] OR nasal[Title/Abstract]) AND ("nasal aging"[Title/Abstract] OR "nasal ageing"[Title/Abstract] OR "aging nose"[Title/Abstract] OR "ageing nose"[Title/Abstract] OR "age-related"[Title/Abstract] OR aging[Title/Abstract] OR ageing[Title/Abstract]) AND (skin[Title/Abstract] OR "soft tissue"[Title/Abstract] OR "soft-tissue"[Title/Abstract] OR cartilage[Title/Abstract] OR framework[Title/Abstract] OR ligament*[Title/Abstract] OR morphology[Title/Abstract] OR skeletal[Title/Abstract])	133
3. Autologous grafts and long-term contour outcomes	rhinoplasty[Title/Abstract] AND ("autologous	105

	cartilage"[Title/Abstract] OR "autogenous cartilage"[Title/Abstract] OR "autologous graft"[Title/Abstract] OR "autogenous graft"[Title/Abstract] OR "cartilage graft"[Title/Abstract] OR "cartilage grafts"[Title/Abstract] OR "costal cartilage"[Title/Abstract] OR "auricular cartilage"[Title/Abstract] OR "septal cartilage"[Title/Abstract]) AND ("long-term"[Title/Abstract] OR "long term"[Title/Abstract] OR resorption[Title/Abstract] OR absorption[Title/Abstract] OR warping[Title/Abstract] OR deformation[Title/Abstract] OR displacement[Title/Abstract] OR migration[Title/Abstract] OR "contour irregularity"[Title/Abstract] OR "contour irregularities"[Title/Abstract] OR "graft visibility"[Title/Abstract] OR complication[Title/Abstract] OR complications[Title/Abstract])	
4. Preservation and camouflage techniques	rhinoplasty[Title/Abstract] AND ("preservation rhinoplasty"[Title/Abstract] OR "dorsal preservation"[Title/Abstract] OR "push down"[Title/Abstract] OR "push-down"[Title/Abstract] OR camouflage[Title/Abstract] OR "diced cartilage"[Title/Abstract] OR fascia[Title/Abstract] OR "temporalis	204

	fascia"[Title/Abstract] OR "temporal fascia"[Title/Abstract] OR "fascia lata"[Title/Abstract] OR AlloDerm[Title/Abstract] OR "soft tissue augmentation"[Title/Abstract] OR "soft-tissue augmentation"[Title/Abstract])	

Note: The original PubMed searches were conducted from database inception through 30 June 2026 using combinations of the predefined search terms. The exact query strings were not prospectively archived. For reporting transparency and reproducibility, the Boolean strategies shown here were reconstructed from the original search domains and terms. The record counts shown are the counts obtained during the original searches and were not recalculated from the reconstructed strings. The reconstructed strategies were not used to retrospectively alter the original screening counts.

Screening summary: 463 PubMed records were identified across the four original searches. After removal of 47 duplicate records, 416 unique records remained. Following title screening, 293 records underwent title and abstract screening, and 205 publications were retained for further narrative assessment. Google Scholar and reference-list searching were supplementary and were not included in the PubMed record count.
